# Pepsin promotes laryngopharyngeal neoplasia by modulating signaling pathways to induce cell proliferation

**DOI:** 10.1371/journal.pone.0227408

**Published:** 2020-01-15

**Authors:** Kai Niu, Chunjie Guo, Shiyong Teng, Dandan Zhou, Shuyuan Yu, Wanzhong Yin, Ping Wang, Wei Zhu, Maoli Duan

**Affiliations:** 1 Department of Otolaryngology Head and Neck Surgery, the First Hospital of Jilin University, Changchun, PR China; 2 Department of Radiology, the First Hospital of Jilin University, Changchun, PR China; 3 Department of Anesthesiology, the First Hospital of Jilin University, Changchun, PR China; 4 Department of Clinical Science, Intervention and Technology, Department of Otolaryngology Head and Neck Surgery, Karolinska University Hospital, Karolinska Institutet, Stockholm, Sweden; Columbia University, UNITED STATES

## Abstract

Pepsin plays an important role in laryngopharyngeal reflux (LPR), a risk factor for the development of hypopharyngeal squamous cell carcinomas (HPSCC). However, the role of pepsin in HPSCC is not clear. We show by immunohistochemistry that pepsin positivity occurs in a significant proportion of human primary HPSCC specimens, and in many cases matched adjacent uninvolved epithelia are negative for pepsin. Pepsin positivity is associated with nodal involvement, suggesting that pepsin may have a role in metastasis. Treatment of FaDu cancer cells with pepsin increased cell proliferation, possibly by inducing G1/S transition. We also observed significant changes in expression of genes involved in NF-kappaB, TRAIL and Notch signaling. Our data suggest that pepsin plays an important role in HPSCC and that targeting pepsin could have potential therapeutic benefits.

## Introduction

Hypopharyngeal squamous cell carcinomas (HPSCC) have the worst prognosis among head and neck squamous cell cancers (HNSCC), likely because patients are already presented with late stage disease at time of diagnosis [1]. An important risk factor for the development of extra-esophageal cancers is laryngopharyngeal reflux (LPR) [2], in which pepsin is believed to have an important role [3–5]. In a case-control study conducted by Sereg-Bahar and colleagues [6], total pepsin in saliva of patients with HNSCC was found to be significantly higher than that of control subjects. No significant differences in pH were observed, suggesting that nonacidic pepsin reflux is associated with HNSCC. Pepsin is taken up by laryngeal epithelial cells by receptor-mediated endocytosis at neutral pH and detected in intracellular vesicles such as Golgi bodies of low pH [7]. In vitro studies have shown that pepsin can induce a dose- and time-dependent increase in proliferation of hypopharyngeal cells in parallel with changes in expression of microRNA and genes known to be involved in tumorigenesis [8]. Treatment of cells with nonacid pepsin also increased anchorage-independent growth and migration, demonstrated by an increase in colony formation [9].

Taken together, these data strongly suggest that chronic exposure to pepsin promotes the development of laryngopharyngeal cancer.

The role of pepsin in laryngopharyngeal carcinogenesis remains unclear. Reflux esophagitis is associated with the progression of Barrett’s metaplasia to esophageal adenocarcinomas [10, 11], with a potential involvement of NF-κB signaling [12]. It has been speculated that refluxed pepsin and bile stimulate the release of inflammatory cytokines from esophageal squamous cells, resulting in recruitment of lymphocytes to the submucosa and subsequently to the luminal surface of the esophagus [10]. These events lead to activation of NF-κB signaling in Barrett's cells, enabling these cells to resist apoptosis in spite of DNA damage [10]. Treatment of Fadu cells with pepsin in a nonacidic environment induced the expression of several pro-inflammatory cytokines and receptors, including those involved in inflammation of esophageal epithelium in response to reflux [13].

In this study, we evaluated primary human HPSCC and adjacent noninvolved tissues for pepsin staining by Immunohistochemistry (IHC). We also investigated the in vitro effect of nonacidic-pepsin on signal transduction pathways and cellular functions in an effort to understand the role of pepsin in HPSCC pathogenesis.

## Methods

### 1. Patients and tissue samples

Primary HPSCC specimens were obtained from 70 patients at the first affiliated hospital of Jilin University (Changchun, China) between August 2013 and August 2016. The inclusion criteria for patient selection were: 1) previously untreated hypopharyngeal cancer, 2) histologically confirmed squamous cell carcinoma, and 3) no distant metastasis at the initial visit. Of the 70 patients, 68 were men and 2 were women. The median age of patients was 54.5 years (range, 45–76 years). Tumor samples were obtained from radical resection of the HPSCCs. Pathological evaluation indicated that these tumors ranged from stage I to stage IV. Uninvolved tumor-adjacent tissues were obtained 1cm away from the cancers and were confirmed as non-cancerous by a certified pathologist. For negative control samples, we used mucosa from 4 pediatric patients who received tonsillectomy and had no clinical signs or symptoms of LPR as determined by the reflux finding score (RFS) and the Reflux Symptom Index (RSI) questionnaire. For positive controls, normal stomach tissues were obtained from patients with esophageal cancer. All tissue samples were formalin fixed and paraffin embedded (FFPE). This study was approved by the independent ethics committee of the Jilin University under project number 2013/091 (June 2013). Written consents were obtained from all patients involved in the study.

### 2. Immunohistochemical analysis of pepsin expression

IHC was performed using the SP-kit (Bioss, Beijing, China) following the instructions of the manufacturer. Briefly, 3.0 μm sections were placed on glass slides, dewaxed, and rehydrated. Antigen retrieval was performed in citrate-buffered saline using a microwave oven for heating. Endogenous peroxidase activity was blocked by incubating sections in 3% H_2_O_2_ for 15 min at RT. Sections were blocked in 5% goat serum for 10 min and incubated with rabbit polyclonal anti-pepsin primary antibody (1:100, EIAab Science, China) overnight at 4°C. Sections were washed 3 times in PBS, incubated with biotinylated goat anti-mouse secondary antibody for 20min, and then with streptavidin-HRP conjugate for 20min. Sections were thoroughly washed in PBS and incubated with DAB substrate for 10 min for signal detection. Negative and positive control samples (see above) were routinely included in the IHC analysis.

Table 1 outlines the scoring system used to assess pepsin staining in primary HPSCC specimens. The scoring was based on a combination of staining intensity and percentage of pepsin-positive tumor cells. Specimens with a point score of 3 or higher were considered as pepsin positive.

**Table 1:** Scoring system used for evaluation of pepsin staining in primary HPSCC specimens.

Staining Intensity	Points	Staining Proportion	Points
No staining	0	0%	0
Low	1	<10%	1
Moderate	2	10–29%	2
High	3	30–59% 60–100%	3 4

### 3. Cell culture

Human HPSCC FaDu cells (ATCC, Manassas, VA) were grown in Minimum Essential Medium–Eagle with Earle’s Balanced Salt adjusted to 1.5 g/L sodium bicarbonate. The growth medium was supplemented with 0.1 mM nonessential amino acids, 1.0 mM sodium pyruvate, and 10% fetal bovine serum (ATCC). Cultures were incubated at 37°C under 5% CO_2_, and sub-cultured when reached 70% confluence.

#### Cell viability

FaDu cells were treated with porcine pepsin (0.2 or 0.4ug/ml; Sigma-Aldrich, St. Louis, MO) for 0.5, 1, 2 or 4 hours in 96-well plates. Treatments were performed at pH7, and 5 technical replicates were included for each treatment condition. After the treatment, cells were washed with PBS and grown in fresh growth media. Cell viability was determined using a CCK-8 solution (Beyotime Biotechnology, China) 24 hours after treatment. At a pre-determined assay time point, a 10% (v/v) CCK-8 solution was added to each well and incubated for 1 hour. Absorbance at 450 nm was measured in a Bio-Rad 480 microplate reader (Bio-Rad Laboratories, Hercules, USA). To determine the effect of irreversibly inactivated pepsin on cell proliferation, pepsin was inactivated at pH 8.0 for 15 minutes at 37°C and returned to pH to 7.0 for cell treatments.

#### Flow cytometry

FaDu cells were treated with porcine pepsin (0.2 mg/mL, pH 7.0, 37°C) for 0.5 or 1 hour, washed three times in fresh media, and incubated for a further 24 hours in complete growth media. Cells were fixed in 70% ethanol, incubated with a propidium iodide/Triton X-100 staining solution containing RNase A (50μg/ml PI +200μg /mL RNase A), and assessed for cell cycle distribution using the Click-iT EdU Alexa Fluor 647 Flow Cytometry Assay Kit (Beyotime Biotechnology, China) according to manufacturer’s instructions.

#### Confocal microscopy of FaDu cells treated with Cy3-labeled pepsin

Cy3-labeled pepsin was supplied by Bioss antibodies (Woburn, MA, USA). FaDu cells were treated with Cy3-labeled porcine pepsin (0.2ug/mL; Bioss Antibodies (Woburn, MA, USA) at 37°C for 30 minutes in pH 7 growth media. Cells were washed and incubated for a further 24 or 36 hour in complete growth media. Cells were then washed with PBS and incubated with Lyso-Tracker red (50 nM; Beyotime Biotechnology, China) in DMEM containing 10% FBS for 60 min at 28°C. Cells were washed, fixed, and stained with 40, 6-diami-dino-2-phenylindole (DAPI). Following washes in PBS, cells were analyzed by confocal microscopy (Fluo-View FV1000; Olympus, Japan).

### 4. Human Signal Transduction PathwayFinder

The Human Signal Transduction PathwayFinder^™^ RT2 Profiler^™^ PCR Array (PAHS-014Z, Qiagen, Frederick, MD, USA) was used to evaluate the expression of a panel of 84 genes representative of ten different signal transduction pathways, in FaDu cells treated with pepsin. Total RNA was isolated from pepsin treated Fadu cells and control cells using the Qiagen RNeasy Mini Kit, following manufacturer’s protocol. RNA was quantified using the Nanodrop 2000 (Gene Company Limited, Hong Kong, China), and quality was assessed based on the integrity of 18 S and 28 S ribosomal RNA bands in 1% agarose gels. First-strand cDNA was mixed with 2 × RT2 SYBR Green qPCR Master Mix and ddH_2_O. qPCR was performed in the Applied Biosystems (ABI) 7500 system using the following conditions: 95°C for 10 min followed by 40 cycles of 95°C for 15 sec and 60°C for 1 min. Each array contained five independent housekeeping genes (Actb, B2m, Hprt1, Ldha and Rplp1) that were used for data normalization.

### 5. Semi-quantitative analysis of immunofluorescent staining

FaDu cells were grown on slides, fixed in 4% paraformaldehyde in PBS for 15 min at RT and washed with PBS. Cells were permeabilized with 0.2% Triton X-100 in PBS for 10 min. Cells were blocked with 5% goat serum for 1 hour, and incubated with rabbit polyclonal anti-p21 (1:100; Bioss Antibodies), rabbit polyclonal anti-C-Myc (1:100; Bioss Antibodies), rabbit monoclonal anti-NFκB p65 (1:100, Abcam) overnight at 4°C. The next day cells were washed 3 times with PBS, and incubated with goat Alexa 555 conjugated anti-rabbit IgG (1:400, Abcam) for 1 hour at room temperature in the dark. Cells were mounted in 70% glycerol and images were taken by laser confocal microscopy (Fluo-View FV1000; Olympus, Japan).

Detection of the fluorescent intensity (FI) of FaDu cells stained with anti-p21 or anti-C-myc antibodies were preformed under a laser scanning confocal microscope. Positive signals were analyzed as mean fluorescent intensity (MFI) using the FV10-ASW 4.0 software (Fluo-View FV1000; Olympus, Japan). In brief, 100 cells from each treatment group were analyzed in a blinded manner. All images were captured under the same camera settings.

### 6. Western analysis

FaDu cells grown in 6-well plates were treated with pepsin (0.2 mg/mL) in pH7.0 for 30 min. Levels of phosphorylated of IκB and p65 were evaluated by Western analysis. Rabbit polyclonal anti- p65, anti-phospho-p65, anti-IκB, and anti-phospho- IκB antibodies were purchased from Abcam (Cambridge, UK). The secondary antibodies were Goat anti-rabbit antibodies conjugated with horseradish peroxidase purchased from Abcam (Cambridge, UK). Signals were visualized by ChemiDoc XRS+ using the Image LabTM Software (Bio-Rad Laboratories, Munich, Germany). Protein levels were quantified by scanning densitometry.

### 7. Statistical analysis

Proliferation assays. Data from five biological replicates for dose-response experiments were analyzed by one-way analysis of variance and Tukey multiple comparisons post-test. Data are expressed as mean±standard deviation. Microarray data was normalized against the house keeping genes by calculating the ΔCt for genes of interest. Fold changes in expression levels were analyzed using the RT2 PCR array data analysis web portal version 3.5 (http://pcrdataanalysis.sabiosciences.com/pcr/arrayanalysis.php). Genes with more than a 1.5-fold change in expression levels between pepsin-treated and control groups were considered significant.

## Result

### 1. Pepsin staining in primary HPSCC tumors and adjacent epithelia

**Fig 1:**
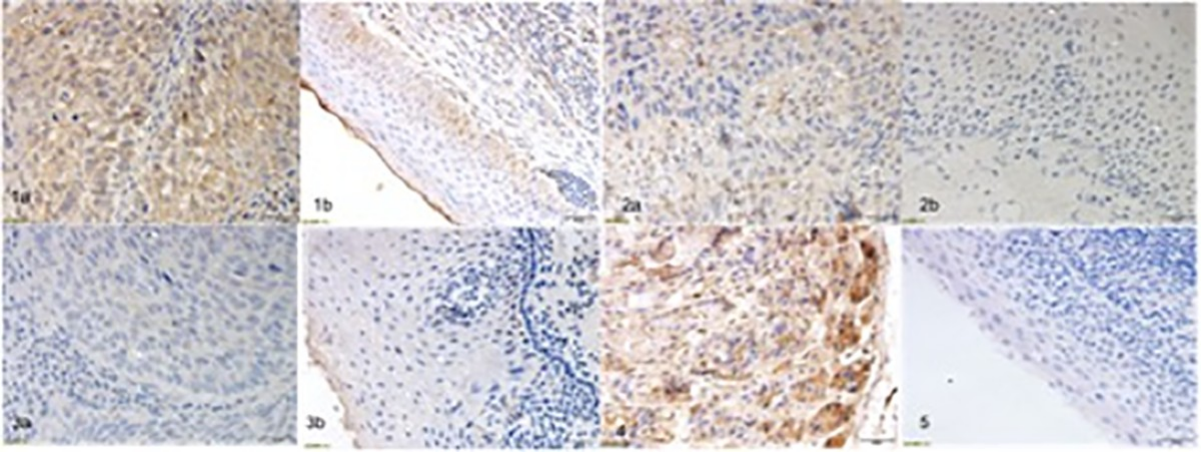
Pepsin staining in primary HPSCC tumors and matched adjacent tissues. Pepsin stained positively in HPSCC tumor 1a and matched adjacent tissue 1b. There was no pepsin in HPSCC tumor 3a and matched adjacent tissue 3b. Tumor 2a was positive but adjacent tissues 2b was negative, for pepsin staining. Gastric oxyntic mucosa (4) showed strong pepsin staining while tonsil (5) showed negative pepsin staining.

Levels of pepsin protein in human primary HPSCC and corresponding uninvolved adjacent tissues were assessed by IHC. As shown in Fig 1, gastric oxyntic mucosa and tonsil tissues were used as positive and negative controls, respectively. Pepsin staining was localized to the cytoplasm of tumor cells as well as adjacent epithelial cells (Fig 1). Table 2 summarizes pepsin staining in primary HPSCC and matched uninvolved adjacent epithelium specimens. Of the 70 paired specimens, 21 had positive pepsin staining in both tumor and adjacent tissues. We observed 18 cases where pepsin was detected in the tumor but not in the adjacent epithelium. Pepsin was not present in both tumor and adjacent tissues in 31 cases.

**Table 2:** Summary of pepsin staining in matched tumor and matched adjacent uninvolved tissues.

	Pepsin (+)(%)	Pepsin (-)(%)	total	p
Primary tumor	39(55.7%)	31(44.3%)	70	0.002
Tumor-adjacent tissue	21(30.0%)	49(70.0%)	70	

### 2. Pepsin is associated with nodal metastasis in HPSCC patients

To understand the relevance of pepsin positivity in HPSCC pathogenesis, correlative studies were performed using available clinical and pathological data. As summarized in Table 3, there was no association between pepsin positivity and alcohol and tobacco consumption. There was also no association between pepsin staining and tumor stage and grade. On the other hand, we observed a statistically significant association between pepsin positivity and nodal involvement (P = 0.027, χ2 test). Whereas 35% of HPSCC patients without nodal metastasis were presented with pepsin positive tumors, 64% of tumors from patients with nodal metastasis were positive for pepsin.

**Table 3:** Correlation between pepsin and clinical and pathological characteristics of HPSCC.

Clinical Characteristics	Overall	Pepsin expression		P Value
		positive	negative	
Alcohol consumption				
Daily	53	28	25	0.391
Rare/never	17	11	6	
Tobacco exposure				
Smoker	55	31	24	0.834
Nonsmoker	15	8	7	
Staging and Grading				
Tumor stage (pathological)				
T1/2	16	7	9	0.273
T3/4	54	32	22	
Nodal stage (pathological)				
N0	20	7	13	0.027*
N1–3	50	32	18	
Grading				
G1/2	23	13	10	0.924
G3/4	47	26	21	

*P<0.05.

### 3. Pepsin induced G1/S transition resulting in increased proliferation of FaDu cells

**Fig 2:**
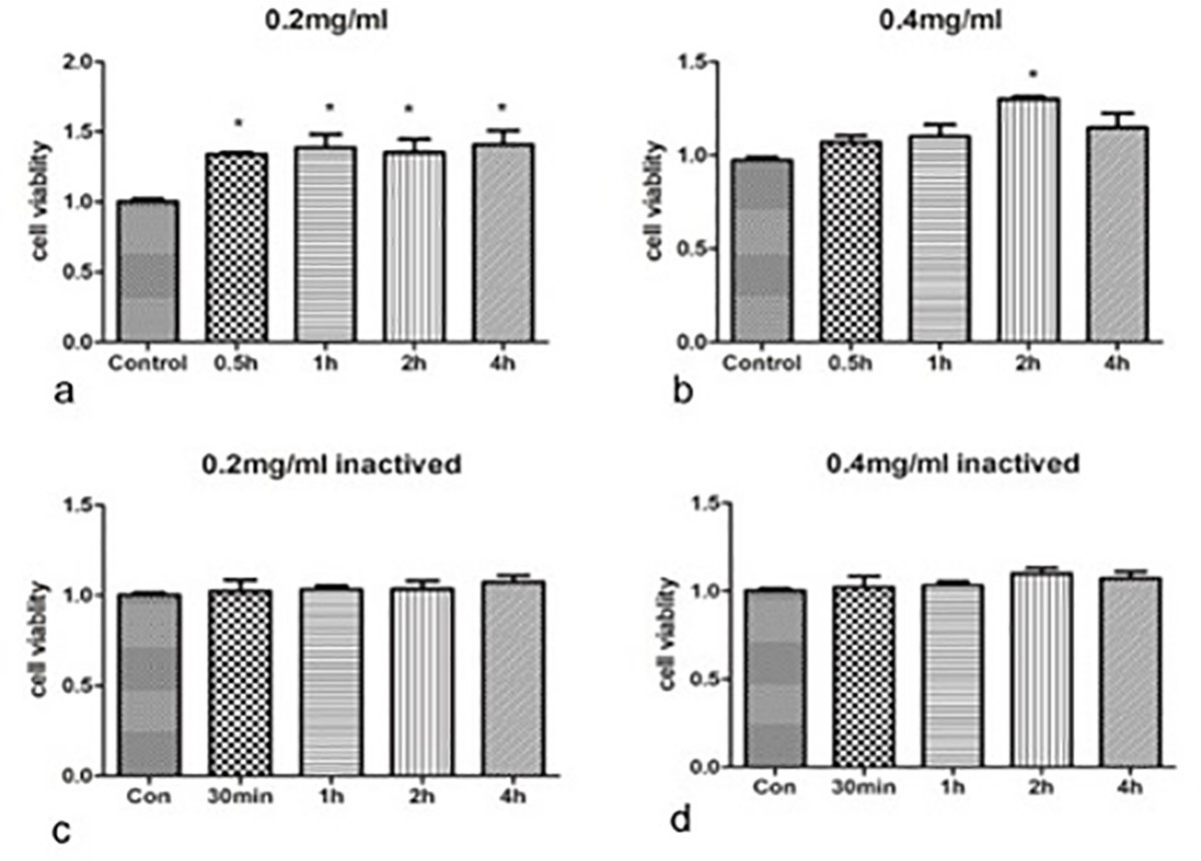
Pepsin increases the growth and proliferation of FaDu cells. FaDu cells were treated with pepsin for the indicated length of time and cultured in fresh complete growth media for 24 hours before analysis. (a) FaDu cells treated with pepsin at a concentration of 0.2mg/ml at pH 7 for 30 min and incubated in fresh media for 24 hours at 37°C. (b) Treatment with pepsin at a concentration of 0.4 mg/mL. (c, d) Treatment with irreversibly inactivated pepsin. Data are from five biological replicates. Bar graphs show mean ± standard deviation. Dose-response data were analyzed by one-way analysis of variance and Tukey multiple comparisons post-test. Time-response data were analyzed by two-way analysis of variance and the Bonferroni multiple comparisons post-test. *P < 0.05.

Treatment of FaDu cells with pepsin at a concentration of 0.2 mg/ml (pH 7.0) for 30 mins resulted in a significant increase in cell number (Fig 2A). At this concentration of pepsin, extending the duration of treatment to 1–4 hours did not result in further increase in cell number. When the concentration of pepsin was increased to 0.4 mg/mL (pH 7), treatment of FaDu cells for 2 hours also resulted in a significant increase in cell number (Fig 2B). However, there was no effect on cell number when cells were treated for 30 min, 1 hour, or 4 hours. Treatment of FaDu cells with irreversibly inactivated pepsin under the same conditions had no effect on cell number (Fig 2C and 2D). Therefore, subsequent experiments were performed at a pepsin concentration of 0.2 mg/ml.

**Fig 3:**
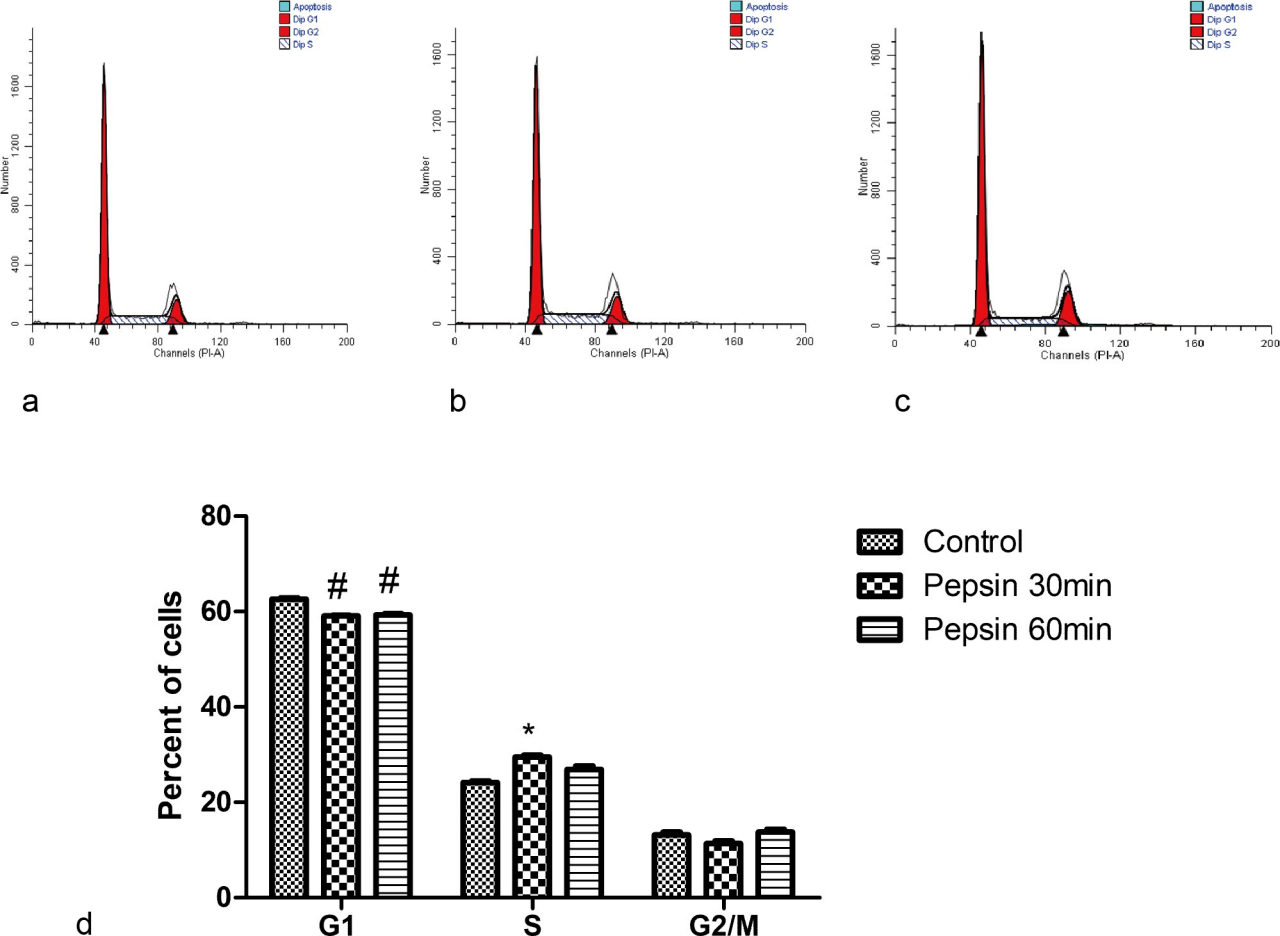
Cell cycle distribution of FaDu cells in response to pepsin treatment. Cells were fixed, stained with propidium iodide, and analyzed by flow cytometry. (a) control, (b) pepsin (0.2mg/ml) treatment for 30 min, and (c) pepsin treatment (0.2mg/ml) for 1 hour at 37°C. (d) Summary of data. Data are from five biological replicates and presented as mean ± standard error of the mean. Statistical analyses were performed by one-way analysis of variance and Tukey multiple comparisons post-test. *P<0.05.

To further investigate the effect of pepsin on cell proliferation, we determined cell cycle distribution of FaDu cells following pepsin treatment. Cells were treated with 0.2 mg/ml (pH 7) of pepsin for 30 mins or 1 hour, fixed, and stained with propidium iodide for analysis by flow cytometry. When cells were treated with pepsin for 30 min, we observed a significant increase in percentage of cells entering the S phase and a corresponding decrease in percentage of cells in the G1 phase in comparison with control cells (P* < 0.05, Fig 3). Treating cells with pepsin for 1 hour resulted in a significant decrease in percentage of cells in the G1 phase (P < 0.05), but there was no significant difference in percentage of cells in the S or G2/M phase when compared to controls (Fig 3)

### 4. Involvement of endosome/.lysosome in pepsin intracellular reactivation

**Fig 4:**
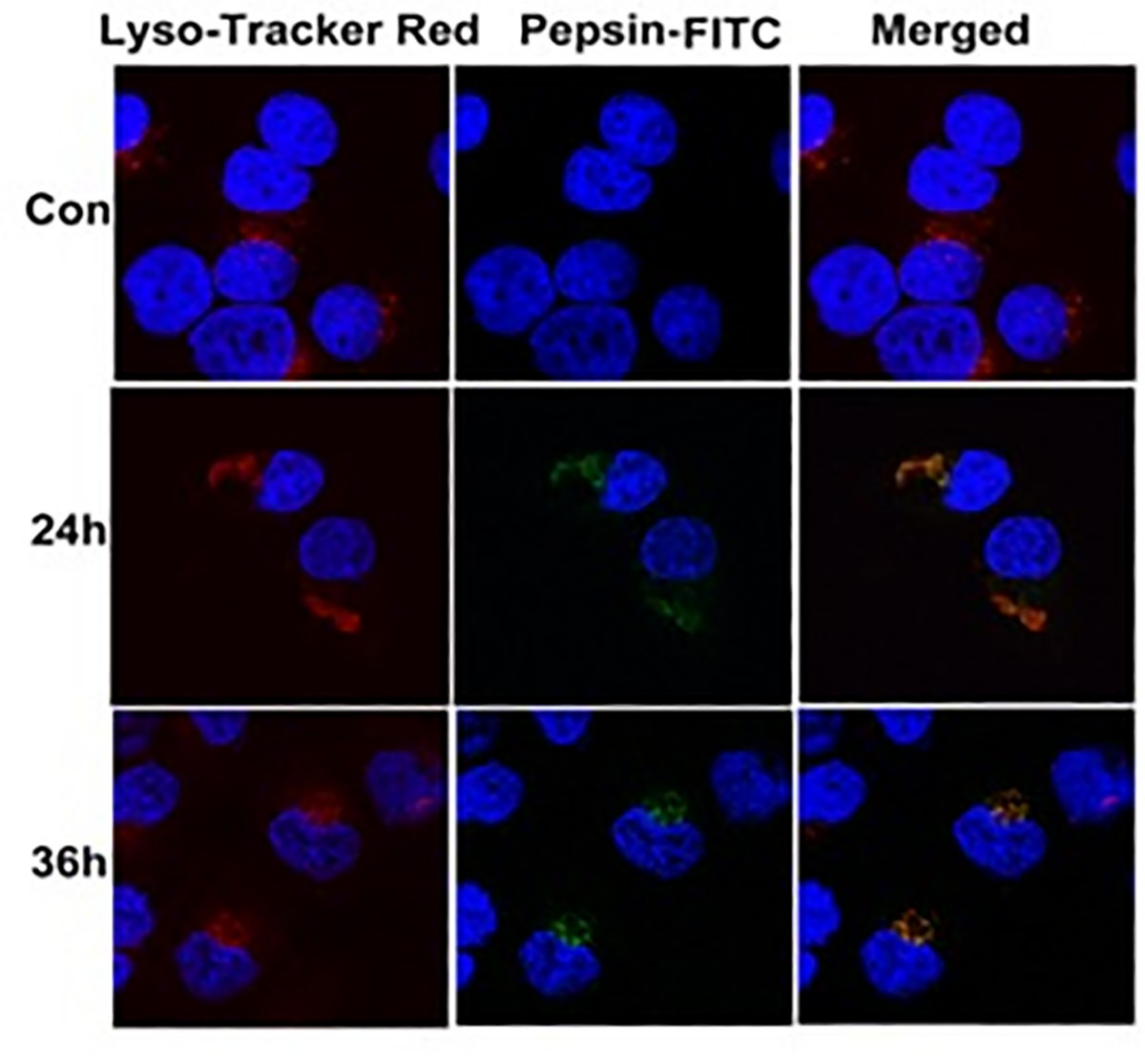
Localization of pepsin to the lysosomes of FaDu. FaDu cells were treated with Pepsin-Cy3 for 0.5 h, washed and incubated for 24 hours or 36 hours. After labeling with Lyso-tracker red, cells were stained with DAPI and visualized under a confocal microscope. Pepsin taken up by cells mainly localized to the lysosomes at 36 hours post-treatment.

To investigate a potential involvement of the endosomes in pepsin reactivation, pepsin-treated FaDu cells were stained with Lysotracker red to track acidic organelles. We observed co-localization of the Lysotracker red and pepsin, consistent with localization of pepsin to the lysosome (Fig 4). Pepsin remained localized to the lysosome up to 36 hours post-treatment (Fig 4).

### 5. Gene expression readouts of signaling pathways in pepsin-treated FaDu cells

To investigate the effects of pepsin on signaling pathways, we used the Human Signal Transduction Pathway Finder RT^2^ Profiler PCR Array to profile pepsin-treated and control FaDu cells. Expression of 84 genes were evaluated to provide readouts for a range of signaling transduction pathways (S1 Appendix). Our experiments confirmed that pepsin treatment resulted in a >1.5-fold increase in expression of TNF-α and BCL2A1 compared to control cells (P<0.05, Table 4). In contrast, treatment with pepsin resulted in a 1.93-fold and 2.83-fold decrease in expression of TNFSF10 and HES5, respectively (P<0.05, Table 4).

**Table 4:** Genes that were up- or down-regulated by >1.5-fold in Fadu cells treated with pepsin (0.2 mg/mL; pH7; 30 minutes).

Gene Symbol	Gene ID	Fold Change	P value	Annotated Gene functional
		(pepsin vs control)		
HES5	NM_001010926	-2.83	0.02	Transcriptional repressor of genes that require a bHLH protein for their transcription. Plays an important role as neurogenesis negative regulator.
TNF	NM_000594	3.5	0.03	This gene encodes a multifunctional proinflammatory cytokine that belongs to the tumor necrosis factor (TNF) superfamily. This cytokine is mainly secreted by macrophages. It can bind to, and thus functions through its receptors TNFRSF1A/TNFR1 and TNFRSF1B/TNFBR.
TNFSF10	NM_003810	-1.93	0.04	The protein encoded by this gene is a cytokine that belongs to the tumor necrosis factor (TNF) ligand family. This protein preferentially induces apoptosis in transformed and tumor cells, but does not appear to kill normal cells although it is expressed at a significant level in most normal tissues.
BCL2A1	NM_004049	2.24	0.02	This gene encodes a member of the BCL-2 protein family. The proteins of this family form hetero- or homodimers and act as anti- and pro-apoptotic regulators that are involved in a wide variety of cellular activities such as embryonic development, homeostasis and tumorigenesis. This gene is a direct transcription target of NF-kappa B in response to inflammatory mediators.

### 6. Pepsin treatment activates NF-κB signaling in FaDu cells

**Fig 5:**
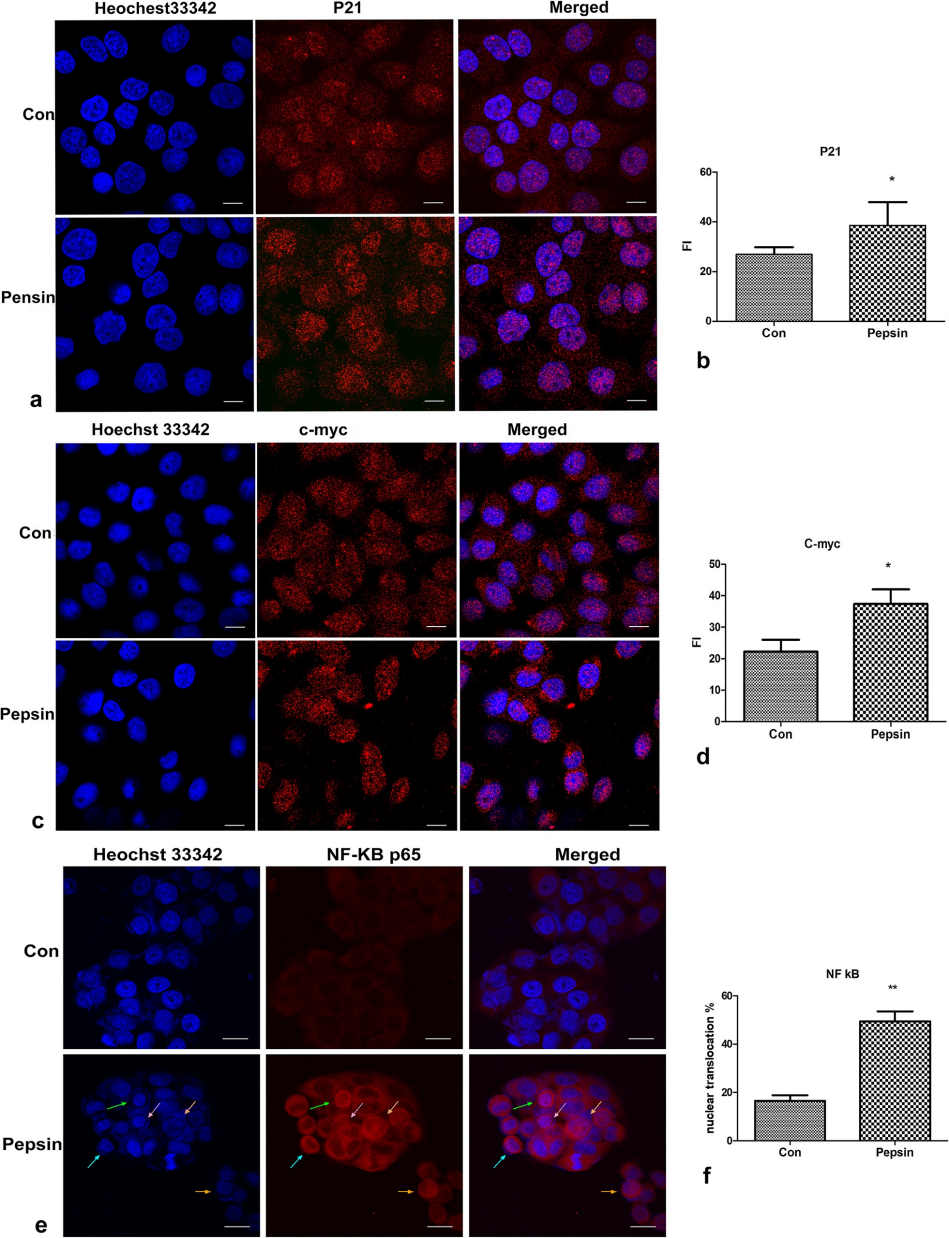
P21, C-Myc, and p65 expression in FaDu cells treated with pepsin. Cells were visualized by confocal microscopy (left panel, magnification = 400X). Quantifications are presented on the right panel. Control and pepsin treated cells were analyzed for expression of p21 (a, b), c-Myc (c, d), and NF-κB p65 (e, f). Arrows in (e) indicate nuclear translocation of NF-κB p65 protein. Hoechst 33342 was used to identify nuclei. Triplicate samples of 100 cells were scored, and data are presented as mean percentage ± SD. * P < 0.05, ** P <0.01.

**Fig 6:**
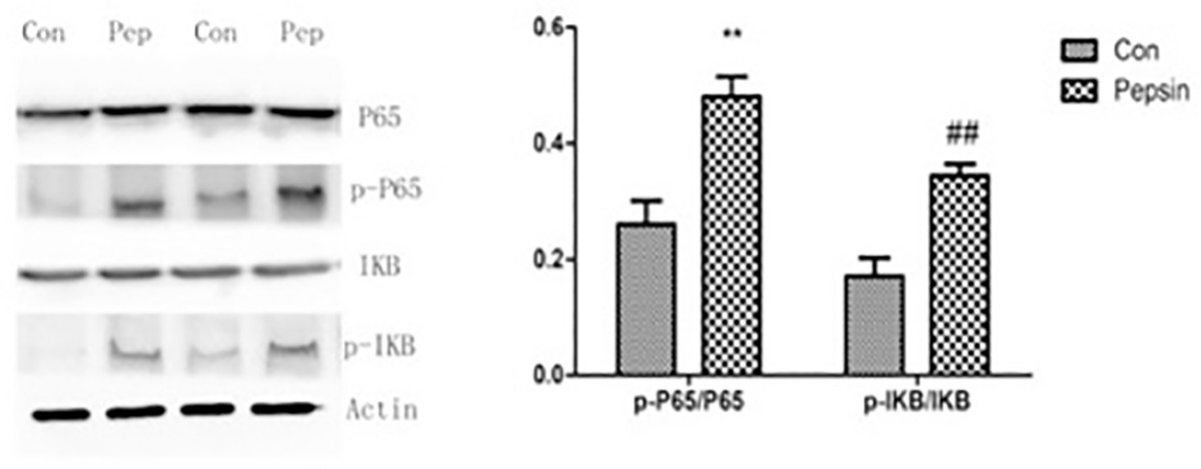
Pepsin induced phosphorylation of P65 and IκB. Protein levels were semi-quantitatively analyzed by scanning densitometry. Ratios of phosphorylated to total proteins are presented on the right panel. **P < 0.05, ^##^P < 0.05.

To confirm our PCR array results, we used immunofluorescent staining and Western blotting to assess the protein levels of selected genes in FaDu cells treated with pepsin in comparison to control. Semi-quantitative analysis of immunofluorescent signals confirmed that treatment of FaDu cells with pepsin induced the protein levels of NF-κB p65, p21 and c-Myc (Fig 5, p < 0.05). We further investigated the effect of pepsin on NF-κB signaling by assessing levels of phosphorylated p65 and IκB in FaDu cells treated with 0.2 mg/mL of pepsin for 30 minutes. Western analysis showed that treatment with pepsin induced levels of phospho-p65 and phospho- IκB, consistent with activation of NF-κB signaling (Fig 6, P < 0.05).

## Discussion

A high prevalence of LPR has been reported in patients with HPSCC, but whether it contributes to cancer growth and metastasis remains unclear. Pepsin is considered as an important clinical marker for LPR when detected in the upper aerodigestive tract [14–16]. Pepsin induces the proliferation of hypopharyngeal cancer cell lines in a dose- and time-dependent manner [8], and has also been shown to inhibit apoptosis [9]. Using a hamster buccal model, Adams et al [17] demonstrated that chronic exposure to pepsin together with DMBA resulted in a higher incidence of dysplasia than DMBA treatment alone. Results from this study confirm a role for pepsin in the promotion of HPSCC development and further suggest that this may driven in part by activation of NF-κB signaling.

We evaluated pepsin expression in 70 primary human HPSCC specimens and matched adjacent uninvolved epithelial tissues by IHC. Pepsin was detected in 39 of the 70 HPSCC specimens (Fig 1, Table 1). Of the 39 pepsin-positive HPSCC specimens, pepsin was also detected in the adjacent epithelial in 21 of the cases. However, in 18 cases pepsin was detected in HPSCC but not in adjacent epithelial samples. Importantly, pepsin expression in primary HPSCC was associated with nodal involvement, suggesting that pepsin may play a role in metastasis. Due to unavailable clinical follow-up data at this time, we were unable to determine whether pepsin positivity is associated with disease-free or overall survival.

Our in vitro studies showed that treatment with pepsin at concentrations of 0.2mg/ml and 0.4mg/ml for 30 min induced proliferation of the FaDu hypopharyngeal cancer cells. Extending pepsin treatment time did not further increase cell proliferation.

Previous studies showed that pepsin is taken up by hypopharyngeal epithelial cells by receptor-mediated endocytosis at neutral pH and localizes at late endosome and trans-reticular Golgi up to 6hrs post-treatment [18]. We observed that within cells pepsin is localized to the lysosome where the pH is in the acidic range (~pH 4.0) [19], suggesting that the lysosome may be the organelle within which inactive pepsin is reactivated. Together with the observation that irreversibly inactivated pepsin did not induce cell proliferation, we speculate that the enzymatic activity of pepsin has an important role in inducing cell proliferation.

Data from our PCR arrays revealed that pepsin treatment up-regulated NF-κB signaling related genes TNF-alpha, TNFSF10 (TRAIL), and BCL2A1 compared to control. TNF family cytokines trigger a variety of NF-κB-dependent responses that can be specific to both cell type and signaling pathway [20, 21]. The roles of NF-κB in determining chronic inflammation and carcinogenesis have been well demonstrated [22], and both of these functions may be crucial to head and neck carcinogenesis. During the development of HNSCC, NF-κB is frequently up-regulated from premalignant lesions to invasive cancer [23, 24], and has been associated with tumor invasion and metastasis [25]. In an in vitro model of gastroduodenal reflux model, Sasaki et al [26] observed Bcl-2 overexpression and significant transcriptional deregulation of NF-κB–related genes with oncogenic function in hypopharyngeal cancer cells treated with acid/ bile. In another in vitro study performed by Sasaki el al [27], weakly acidic-pepsin (pH 5.0) and neutral-pepsin (pH 7.0) were found could induce mild activation of NF-κB with increase in TNF-α mRNAs in human hypopharyngeal primary cells, that is in accordance with our study, but no oncogenic transcriptional activity was detected in their study. This could be explained that the mild increase of NF-κB activity may be related to stress reaction[27] and in vitro cellular study could not mimic the dynamic events in tissue response to selected ranges of acidified pepsin.

NF-κB and TRAIL signaling pathways are important in the regulation of proliferation and apoptosis. NF-κB has many cellular functions and targeting NF-κB for therapeutic applications may lead to severe side effects. Targeting of TRAIL on the other hand can selectively induce apoptosis in cancer cells without affecting normal cells [28], indicating that TRAIL may be suitable target for anti-cancer therapy [29]. Using a panel of HNSCC cell lines, Ren et al [30] showed that targeting of TRAIL and Smac bypassed NF-κB activation to induce cancer cell death, raising the potential benefit of co-targeting strategies involving TRAIL for treating HNSCC.

We identified three target genes (HES5, HEY1, HEY2) of the NOTCH pathway to be significantly altered at RNA levels in FaDu cells treated with pepsin (Table 4). NOTCH signaling is mediated through binding of ligands (JAG1 and -2, and DLL1, -3, and -4) to the NOTCH receptor (NOTCH1, -2, -3, and -4). We found that the expression of NOTCH1 and JAG1 were not affected by the pepsin treatment. After binding of ligand to NOTCH, γ-secretase complex releases the NOTCH intracellular domain (NICD), which moves to the nucleus, resulting in the transcriptional activation of NOTCH target genes [31]. HES, HEY, CCND1, MYC, BCL-2, and p21 are among a large number of NOTCH target genes [32]. The role of NOTCH signaling in promoting or suppressing the development of HNSCC remains controversial [33]. HES5 has an important role in regulating mammalian neuronal differentiation and maintaining neural stem cells [34]. A recent study showed that HES5 silencing is an early and recurrent event in prostate tumorigenesis [35]. In addition, Upadhyay et al [36] showed that Notch pathway activation is essential for maintenance of stem-like cells in early tongue cancer, and the effect of Notch was enhanced by TNF-alpha [37]. However, Wirth et al [38] found that high levels NOTCH1 mRNA is associated with better survival in HNSCC. Mutations studies in HNSCCs have identified loss-of-function mutations in the NOTCH signaling pathway [39], consistent with the observation that inactivation of canonical Notch signaling drives head and neck carcinogenesis in mouse models of keratinizing HNSCC[40]. Additional studies will be needed to elucidate the exact role of pepsin in modulating the NOTCH pathway.

Our research was performed in a cancer-derived cell line that might responds to pepsin differently compared with normal epithelial cells. This is one major limitation of our research and will be resolved in the future study using cultured primary epithelial cells.

## Conclusion

Results presented in this study suggest that pepsin reflux induces a dose-dependent increase in proliferation of hypopharyngeal cancer cells, and this effect is mediated by the enzymatic activity of pepsin. Although the exact role of pepsin in hypopharyngeal cancer development is not fully understood, our data suggest that NF-κB, TRAIL and NOTCH signaling, representing major mediators of cell proliferation, differentiation and apoptosis, are likely to be involved. The development of pharmacological inhibitors to specifically target pepsin could potentially modulate these signaling pathways and have therapeutic value for treating HPSCC.

## Supporting information

S1 Appendix Gene expression readouts of signaling pathways in pepsin-treated FaDu cells. (XLS)
